# Supplementary figures and images for: Properties of rabies virus phosphoprotein and nucleoprotein biocondensates formed in vitro and in cellulo
Source: PLoS Pathog. 2022 Dec 8;18(12):e1011022. doi: 10.1371/journal.ppat.1011022 (PMC9767369; doi:10.1371/journal.ppat.1011022)

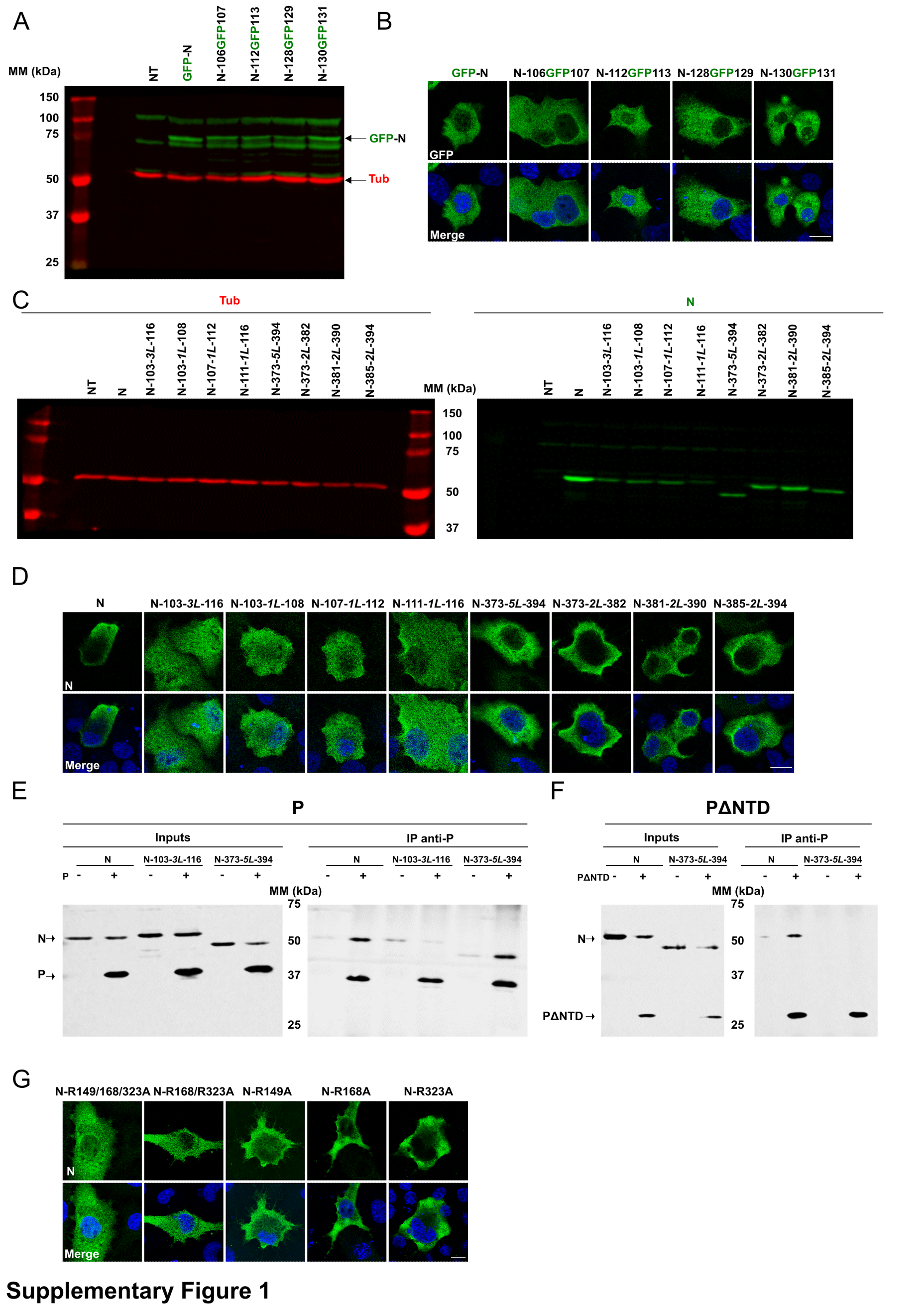

Supplement: S1 Fig — A) and B) BSR-T7/5 cells were transfected for 24 h with pTit plasmids encoding the indicated fluorescent N constructions (GFP-N, N-106GFP107, N-112GFP113, N-128GFP129, N-130GFP131). A) Total expression of the indicated fluorescent N constructions was assessed by western-blot. GFP-N was revealed with a rabbit polyclonal anti-N antibody and tubulin was revealed with a mouse polyclonal anti-tubulin antibody followed by incubation with fluor-800 conjugated anti-rabbit and fluor-680 conjugated anti-mouse IgG. NT: non transfected cells. B) Cellular expression of the indicated fluorescent N constructions was assessed by fluorescence confocal microscopy on fixed cells. N was revealed with a mouse monoclonal anti-N antibody followed by incubation with goat anti-mouse IgG Alexa-488 antibody. C) and D) BSR-T7/5 cells were transfected for 24 h with pTit plasmids encoding the indicated mutants (mutated in loops L1 and L2). C) Total expression of the indicated mutants was assessed by western-blot. N was revealed with a rabbit polyclonal anti-N antibody (right panel) and tubulin was revealed with a mouse polyclonal anti-tubulin antibody (left panel) followed by incubation with fluor-800 conjugated anti-rabbit and fluor-680 conjugated anti-mouse IgG. NT: non transfected cells. D) Cellular expression of the indicated N mutants was assessed by fluorescence confocal microscopy on fixed cells. N was revealed with a rabbit polyclonal anti-N antibody followed by incubation with goat anti-rabbit IgG Alexa-488 antibody. E-F) HEK293T cells were transfected with the pCDNA3.1 plasmids coding the indicated constructs. Cells were lysed and complexes were immunoprecipitated with a cocktail of monoclonal antibodies. Cell extracts (inputs) and immune complexes (immunoprecipitates [IP]) were separated by SDS-PAGE and analyzed by Western blot using polyclonal anti-N and anti-P antibodies. G) BSR-T7/5 cells were transfected for 24 h with pTit plasmids encoding the indicated arginine mutants. Cellular [file ppat.1011022.s001.tif]

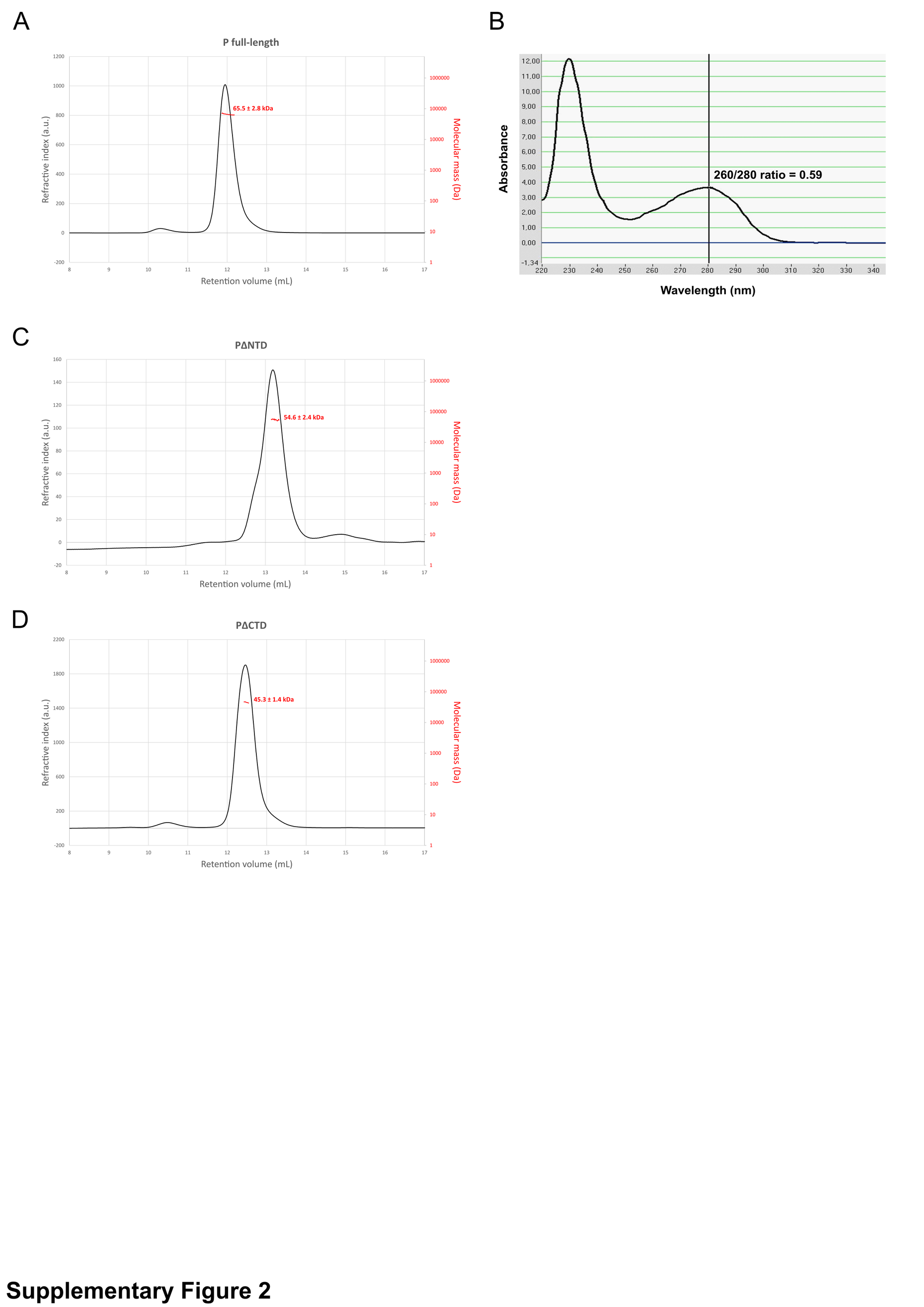

Supplement: S2 Fig — A) SEC-MALS analysis of purified full-length P-Strep. B) Absorbance spectrum of purified P-Strep protein. C) SEC-MALS analysis of purified PΔNTD. D) SEC-MALS analysis of purified PΔCTD. (TIF) [file ppat.1011022.s002.tif]
