# Supplementary material for: Properties of rabies virus phosphoprotein and nucleoprotein biocondensates formed in vitro and in cellulo
Source: PLoS Pathog. 2022 Dec 8;18(12):e1011022. doi: 10.1371/journal.ppat.1011022 (PMC9767369; doi:10.1371/journal.ppat.1011022)
Supplement: Attachment — Submitted filename: Nevers, rabies NP with N.docx [file ppat.1011022.s012.docx]

This paper treats Negri bodies of rabies. The rabies virus makes Negri bodies (NB) that form by liquid-liquid phase separation (LLPS). The ideas for LLPS came from physics and when the authors found that NB for LLPS people from biology and physic could start working together and this science is becoming very hot. A new view of the cell and their viruses.

Gaudin et al have earlier shown that the phosphoprotein (P) is important and now, in this paper, they have shown that the nucleoprotein (N), as N-RNA and N°-P complexes work with P for making the NP. They made fluorescent N’s and found two loops on N that could be used for making NB and also several Arg residues, that probably bind to RNA.

This paper is rather full, full with now ideas and I think the readers of your journal would be happy for trying ideas for in your labs!

I did not any problems, just a few errors in words, spaces between numbers and units. Other wise, I think that this paper would be very good for PLoS Pathogens.

Comments

Lines	210; simultaneously

228; E; Coli

230; 10 µM and next line 5 µM

235; 50 mM

236; 1 µM

237; 50 mM

238; 10 µM

261; E. Coli

270; 25 nM

580; 2 mM

915; 20 mM

927; 20 mM

941; 1 µM
