# Supplementary material for: Properties of rabies virus phosphoprotein and nucleoprotein biocondensates formed in vitro and in cellulo
Source: PLoS Pathog. 2022 Dec 8;18(12):e1011022. doi: 10.1371/journal.ppat.1011022 (PMC9767369; doi:10.1371/journal.ppat.1011022)
Supplement: Attachment — Submitted filename: rebuttal.docx [file ppat.1011022.s013.docx]

We thank the reviewers for their careful reading of the manuscript and for the overall interest they have shown in our work.

Most of their remarks are well-founded and relevant. We have therefore largely taken them into consideration in this revised manuscript version.

We have also tried to respond as best as possible to their remarks by carrying out the additional experiments they suggested.

We have also added new references that were published during the reviewing process of our manuscript (references 30 and 33 on human metapneumovirus and barley yellow striate mosaic virus).

Particularly, we were asked by the editors to focus on the following points:

1) Specific concerns with FRAP experiments including their representation and interpretation

*We have completed the FRAP data. See in particular, our specific answers to reviewer 1.*

2) The difference of NBs obtained with or without infection are not discussed in the text. The authors should calculate the Cdense/Cdiffuse as described the Brangwynne group (Riback et al. Composition-dependent thermodynamics of intracellular phase separation. Nature 581, 209–214 (2020)

*We have quantified the concentrations of P and N in the diffuse and dense phases. Quantifications are presented in a new figure 3 and in a new paragraph in the results section (p11 and 12).*

*This allowed us to demonstrate that the behaviors of some mutants are distinct from those of wild-type N in the cellular minimal system and that they phase separate at higher P and N concentrations.*

*Nevertheless, we observed a positive correlation between the concentrations of P and N in the dilute phases (simultaneously high or simultaneously low), which is unexpected (based on classical models for LLPS). This suggests that in many cases (when the dilute phase concentrations are high), either we are far from equilibrium or the state of the cell has a considerable influence on the phenomenon. In any case, the extraction of thermodynamic parameters from these data seems very difficult if not impossible. This is mentioned in the discussion (p20).*

3) The relevance of data obtained with E.coli-expressed P-protein needs to be confirmed as RABV P-protein does not phase-separate when expressed in cellula but E.coli-expressed purified P-protein phase-separates in vitro. Will purified P-protein expressed in eucaryotic cells also condensate in vitro?

*Using a recombinant baculovirus, we have expressed in Hi-5 insect cells and purified full-length P. This protein phase separates as well as the protein produced in E-Coli (Fig. 4B and 4C)*

4) RNA recruitment into condensates needs to be assessed and possible RNA contamination needs to be ruled out.

*We do not understand the first part of this comment as we presented data showing that RNA was recruited inside P droplets. The association of N^0^P droplets with RNA was obvious to us due to the droplet deformation observed in presence of RNA. Nevertheless, we now show that RNA concentrates inside N^0^P droplets (Figure 7I).*

*We also provide a spectrum of P produced in E.coli in figure S2B. The A260/A280 ratio is 0.59 indicating that the protein is nucleic acids free*

Following are our specific answers to the reviewers comment.

**Part I – Summary**

**Reviewer #1:**

In this article the authors examined the impact of N mutations on the biogenesis of biocondensates in cells and described various properties of biocondensates formed in vitro by P alone or N0-P. Numerous exciting observations are presented in these different models providing insights into the biogenesis and evolution of Negri bodies during infection.

*We thank the reviewer for acknowledging the interest of this work.*

However, these issues are addressed only superficially and a systematic characterization is lacking to strongly support the results and the proposed interpretations.

For the first part, which concerns the study of biocondensates formed in cells upon coexpression of N and P, the authors identify certain residues/regions as important to drive LLPS. For some of these modifications, the authors hypothesize that the ability to induce biocondensate formation depends on binding to RNA or P but do not test these hypotheses.

*We have now characterized the ability of N mutants N-103-3L-116 and N-373-5L-394 used in the cellular minimal system to bind RNA and to bind P. Particularly, we show that N-373-5L-394 still binds full-length P via the NTD of P. Our data also demonstrate that the loop L2 is indeed involved in binding the CTD of P (Fig S1E and S1F).*

*Surprisingly, N-103-3L-116 is unable to bind P despite being well-expressed (S1C, S1D and S1E).*

*The ability of N proteins (and mutants) to bind RNA in a cellular context is difficult to characterize. For this reason, we wrote:*

*“Those data indicate that arginines R149, R168 and R323 play a role in biocondensate formation and* ***suggest*** *that N RNA-binding properties are required for the formation of N-P cytoplasmic inclusion bodies.” (Line 195-197)*

*“This* ***suggests*** *that N-RNA association is important for biocondensates formation in the cellular minimal system.” (Line 410)*

The second part studies the properties of biocondensates formed in vitro from P alone and N0-P complexes. These objects could be of interest as models even if such condensates do not exist in cells. The authors tested the effect of NaCl concentration, RNA addition or liposomes. The experiments are limited to restricted experimental conditions without detailed explorations.

The experiments performed on N0-P condensates are also preliminary. They lead to observations that are mechanistically appealing but should be explored in more depth.

Parts 1 and 2 are independent and are not linked.

*From our point of view and knowledge of the field of LLPS in virology, our work is more complete than previous reports.*

*As examples, we would like to mention the demonstration of the ability of droplets to concentrate RNAs, to exclude certain proteins, to associate with liposomes and also the observation of the deformation of N^0^P droplets in presence of RNAs as well as the liquid-gel transition of P droplets.*

*Furthermore, we draw the readers’ attention to the artifacts induced by the use of antibodies to characterize the structure of such liquid compartments.*

*Additionally, the quantification now performed in the cellular minimal systems has never been done before for any virus and raises questions about the possibility of extracting pertinent parameters from those data.*

*Finally, parts 1 and 2, although independent, are linked because they allow the comparison of 2 minimal systems (a cellular and an acellular one).*

**Reviewer #2:**

The role of condensates in RNA virus replication is a highly active research area that is of interest to virologists, physical biologists and drug discovery professionals. Rabies is an important and interesting virus. Thus, the area covered by this paper is of high topical interest.

A role of liquid condensates, called Negri bodies, in Rabies virus replication was already published. That prior work is appropriately cited in the submission and the authors are careful to distinguish between confirmatory and new findings.

*We thank the reviewer for acknowledging the interest in our work and for mentioning our previous work.*

The experimental work is well executed and analyzed throughout.

*We thank the reviewer for acknowledging the quality of our experimental work.*

Figs 1 reports and characterize a minimal system comprising P and N proteins that is sufficient to assemble into condensates in living cells. This system is useful for probing the biophysics of replication condensate assembly and might find use in drug discovery. As such, it is a significant step forward. An interesting feature is that the P protein turns over rapidly within these condensates while the N protein does not. This is an unusual finding. Its mechanistic basis is not clear, though mutagenesis of N takes some early steps towards provide molecular-level understanding.

Fig 2 investigates condensates in RABV infected cells and confirms the interesting finding that P is highly dynamic while N is not. This figure is in part confirmatory of previous work, but the protein dynamics data are novel and the work is important to provide physiological relevance to the minimal system in Figure 1.

*We would like to mention that the behavior of N in NBs as well as in the minimal system had not been characterized before.*

Fig 3-6 report and characterize a series of pure protein experiments in which P, alone or together with N and RNA, undergoes condensation and phase separation in vitro. Crowding agents are necessary, a positive is that the authors show the condensates assemble with multiple crowding agents. I have mixed feelings about these experiments. On the positive side, it is useful and interesting to have a pure protein reconstitution side to the submission. This is highly complementary to the experiments in living cells and sets the stage for more detailed biochemistry and perhaps drug discovery. On the negative, use of crowding agents such as 5% PEG to induce phase separation has been criticized as potentially unphysiological. Use of crowding agents was common in the early years of the condensate field, but it led to the sense that condensation under artificial crowding conditions, which may be much more crowded than the interior of cells, can be an artifact. The debate over use of crowding agents continues and it is important to be cautious in interpreting experiments that use crowding agents to promote condensation of pure proteins. One control is to use mutants to test whether the molecular requirements for condensate assembly are similar in living cells and a pure protein + PEG system. The submission does a pretty good job at that, which makes the data collected with PEG convincing in terms of in vivo relevance. Given the validation by mutant analysis, mechanistic conclusions can be drawn from the PEG-crowded system, though caution will always be required in interpretation.

*We are also aware of the possible artifacts that are encountered when using crowding agents. This is the reason why we have used several of them (PEG, Ficoll and BSA). All induce LLPS at a P concentration of 1µM (~30 µg/ml).*

One specific concern with Figs 3-6 is that hardening of protein condensates over time can be caused by RNA contamination. It is important that the authors provide documentation that their protein preparations are free of contaminating RNA, which often co-purifies with RNA binding proteins and can be challenging to remove.

*P is not known to bind RNAs with sufficient affinity. Indeed, the P spectrum (Figure S2B) reveals a ratio A260/A280 which is consistent with an absence of RNA contamination.*

Overall, the paper provides significant new information about how RABV P and N proteins assemble intro condensates and the different dynamics of P and N in these condensates. The concepts are not new, the details are new, the experiments are well done. Use of mutants to validate minimal and reconstituted approaches is an important positive of the submission. The systems reported might be useful for anti-viral drug discovery, which is another significant positive. Overall, the work is clearly worth publishing, To this reviewer, the paper is somewhat lacking in information relevant to understanding the precise molecular interfaces involved in condensate assemble and the precise role of condensate biophysics in virus infection. These deficiencies, combined with a lack of conceptual novelty, decrease impact, but it is still a useful paper on an important virus.

*We thank the reviewer for his/her generally positive comments.*

*Concerning the precise molecular interfaces involved in LLPS, we think that they have not been characterized in any system. Some domains have been identified, some residues (such as aromatic residues and arginines likely involved in cation-pi interactions) have been implicated in these processes but we are far from precise molecular interfaces.*

*Of note, beyond the flexible C-terminal loop 2 (which* *binds the C-terminal domain of P when N is associated with RNA) and several arginine residues (involved in RNA binding) which could be suspected to play a role in LLPS, we identified the flexible loop 1 (Residues 104-117) which was not presumed to be involved in this process (nor in the binding to P protein).*

*As already mentioned to reviewer 1, we think that the ability of droplets to concentrate RNAs, to exclude some proteins, to associate with liposomes as well as the deformation of N^0^P droplets by RNAs and the liquid-gel transition were not previously described.*

*We also draw the readers’ attention to the artifacts induced by the use of antibodies to characterize the structure of such liquid compartments.*

*Additionally, the quantification now performed in the cellular minimal systems has never been done before for any virus and raises questions about the possibility of extracting pertinent parameters from those data.*

**Reviewer #3:**

The laboratory of Yves Gaudin had previously shown that Negri bodies, the viral factories specialized for transcription and replication of Rabies virus (RABV), have liquid properties, and are formed by liquid-liquid phase separation (LLPS). They also showed before that the co-expression of RABV nucleoprotein (N) and phosphoprotein (P) in mammalian cells is sufficient to induce the formation of cytoplasmic biomolecular condensates having properties that are like those of NBs. In addition, they showed, using a cellular minimal system which domains in P are essential for the formation of biomolecular condensates. Now they further characterized the mechanisms of RABV P- and N-induced LLPS. Here they describe the characteristics of N- enabling LLPS and the role of P in the formation of biomolecular condensates. They also highlight the physicochemical features of P and N0- P that may explain some of NBs properties and function. The manuscript is very well written, organized, and the study is robust. Nevertheless, this reviewer points to the lack of quantification and measurements that could elucidate the thermodynamics associated to this process, namely the conditions that lead to higher concentration of material inside NBs or in the diffuse phase

These are important measurements that would increase the contribution of the

manuscript to the field of phase separation.

*We thank the reviewer for his/her positive comments.*

*As suggested by the referee, we have now measured the concentrations of fluorescent versions of P and N in the dense and diffuse phase.*

*All the quantifications are presented in the new figure 3 and in a new paragraph on p11-12. Their impact is commented in a new paragraph in the discussion section (p20)*

**Reviewer #4:**

I think that this paper is very nice with many ideas about rabies and all negatve strand RNA viruses. This paper is written by the people that first seen that Negri bodies are "droplets"! Now, they show how the nucleoprotein bind to P to making the droplets. Also, very new, they add negative charged lipids and they form a membrane around the droplet, just what happens in the cell, before making new viruses.

*We thank the reviewer for his/her positive comments.*

**Part II – Major Issues: Key Experiments Required for Acceptance**

**Reviewer #1:**

1. How many times were each of the experiments repeated? The number of experiments performed is never indicated in the figure legends except for fig2d.

*All experiments were performed at least 3 times on independent samples. This is now indicated in the statistical analysis paragraph in the material and methods section.*

2. Many FRAP experiments are presented and raise several questions.

a. The FRAP curves presented are averages of a limited number of data points, almost always less than 10. Some curves are from less than 5 data points (Fig1c 4 or 5 points, Fig3h 3 points, Fig5b 4 or 5 points, fig6e 3 points).

*FRAP can be indeed use to measure the rate of fluorescence recovery. In this case, an accumulation of FRAP curves is required to get an accurate result. The interpretation of such data also requires an appropriate theoretical model (which is not the case here). Here, we essentially used FRAP to discriminate between two extreme situations: recovery or no of the fluorescence during the observation time. However, as requested by the reviewer, we increased the number of FRAP experiments.*

*In figure 1C, we have now 5, 7 and 7 experimental points. Of note, in this panel, the fluorescence recovery of P in the minimal system had already been published by our group (ref 7). The data obtained here with 5 FRAP curves are consistent with our previous report.*

*In figure 4H (previously 3H), we have now 11, 9, 6 and 6 experimental points. The added points do not change our previous conclusions.*

*In figure 6B (previously 5B), there is no curve with less than 5 data points and the difference between recovery curves on fresh and aged droplets is obvious.*

*In figure 7F (previously 6F), we have now 6 points.*

Moreover, the number of independent experiments done for collecting these data is never indicated.

*We apologize for not mentioning that we had worked on at least three independent samples. This is now indicated in the statistical analysis paragraph in the material and methods section.*

b. Some FRAP curves are not shown: Fig1c: FRAP curves for the N-128GFP129 mutant and Fig2e: FRAP curves for subvolumes

*In figure 1C, FRAP curves for the N-128GFP129 mutant are now presented. We also present FRAP curves for the subvolumes in figure 2E.*

c. Fig1c: For the N-128GFP129 mutant, on the images shown the small condensates change suggesting a change of plane during the acquisition which would impede the interpretation of the results. How is this controlled?

*There are sometimes movements of droplets. It is unavoidable. For this reason, we coded a second macro, which allowed to correct the movement of the condensates (and also of the NBs for that matter) in X and in Y. FRAP curves for which there was an obvious movement of the condensates on the Z axis were discarded from the analysis. Furthermore, when we did not observe recovery, we controlled the absence of an inclusion in another z plane near the bleached zone.*

d. The results of the FRAP experiments seem sometimes in contradiction with the images :

-Fig5b, panel 10nt >4h. The signal at 50s seems equivalent to the pre-bleach signal whereas this is not the case on the curves.

*There is no contradiction. There is indeed a variability between droplets (as shown on the error bars especially large in this experiment). This may be due to variability in the aging kinetics of the droplets.*

*From the FRAP curves, the recovery after 50s is of 0.5 +/- 0.3 for droplet subvolumes and of 0.65+/-0.3 for whole droplets. This is consistent with the images that are shown.*

-Fig6f. The curves do not correspond to the images shown. One observes here an important recovery, similar to that observed on the fig3h whole droplet panel <1h, whereas the curves are very different. The conclusion (line 247) "fluorescence recovery is an extremely slow process" seems not to be poorly supported by the images. The curves are only from 3 acquisitions.

*In figure 7f (previously 6f), we have now doubled the number of FRAP curves (6 acquisitions).*

*Even on the incriminated image, it is obvious that after 300s, the intensity of fluorescence is below the prebleached one. Nevertheless, we agree that « extremely slow » was excessive, we had suppressed «extremely ». We also agree that it is not that different from the recovery seen in figure 4h (previously figure 3h). In fact, we did not compare P and N^0^P fluorescence recovery kinetics. In both cases, we observed recovery consistent with the liquid nature of the fresh droplets.*

-Fig5b. Overall in the partial bleaches, it seems that the fluorescence of the whole structure is strongly decreased after the bleach. This aspect is not commented nor taken into account in the analyses.

*It is difficult to bleach a subvolume without impacting the surrounding fluorescence (i.e. that of the entire structure) because the laser beam that bleaches the region has a Gaussian section which implies that the periphery of the targeted area will always be impacted. This is also one of the reasons why we discussed the data qualitatively.*

e. Fig5b: Why the time scales do not go further for RNAs in particular for the condensates examined after 4 h.

The absence of recovery at 50s does not allow to conclude that recovery is abrogated (line 285).

The same type of fluorescence redistribution kinetics is observed for P (fig3h <1h).

*The slowed kinetics of RNA fluorescence recovery in aged droplets are obvious.*

*For 10nt RNA, the t_1/2_ of recovery is below 0.5s in fresh droplets while it is ~30s in aged droplets.*

*For 40nt RNA, the t_1/2_ of recovery is ~3s in fresh droplets while it is superior to 50s in aged droplets. In this case, if there is fluorescence recovery, it is extremely slow. Nevertheless, the reviewer is correct and we have softened our sentence by replacing « even abrogated » by « almost abrogated ».*

*Below is a comparison of the fluorescence recovery of fluorescent P in fresh droplets (t<1h) and of fluorescent 40 nt RNA in aged P droplets (t>4h), shown on the same graph, to convince the reviewer that the redistribution kinetics are distinct.*

3. In the experiments on cells with or without condensate formation, no quantification is provided. The number of cells analyzed and the number of experiments should be mentioned Fig1b Fig1e Fig1g Fig2a, b and c.

*All images presented in the article are representative of at least three independent experiments. This is now specified at the end of the paragraph dedicated to “Immunofluorescence staining and confocal microscopy” in the material and methods section.*

*Concerning figures 1b, 1e and 1g, the formation of condensates is an all or nothing process: either they are detected or they are absent in all the cells.*

*Similarly, in figure 2a, either the mutants associate with NB or not at all.*

*The differences between mutants sometimes reside in the diffuse phase concentrations of P and N, which are now analyzed quantitatively in new figure 3.*

*Concerning figure 2a, we highlighted an artifact of immunostaining which is very general in the field (see for example panels 2B, 7A and Fig 8 in ref 6 from our group but also figures 3 and 6 in Menager et al. PLoS Pathog. 2009: e1000315). We see no need to quantify this artifact.*

*Regarding figure 2c, the number of experiments was already indicated in the previous version. We have now specified in the legend of panel 2d the number of cells considered. “The total number of cells analyzed was 1555 (353 + 407 + 795) for GFP N and 1804 (241 + 615 + 948) for N-106GFP107”*

4. Do the N mutants support transcription-replication activity and inhibit transcription-replication in the presence of wild-type N? Are they incorporated into virions?

*We have not investigated the ability of mutants to support transcription-replication.*

*This would be a complicated task as in BSR-T7/5, there are only ~10% of the cells that are transfected which will not impact the overall level of transcription-replication. This should be analyzed at the single cell level.*

*Nevertheless, the data presented for mutant N-106GFP107 (fig 2c and 2d) indicate that it probably impacts transcription-replication.*

*Finally, we investigated the incorporation of GFP-N chimera into virions. The blot below indicates that there is very little, if any, incorporation. We did not investigate this point further as it is not essential in the manuscript).*

5. Lines 228-241: A complete description of the in vitro P condensation model and its sensitivity to salinity requires phase diagrams

*N2A cells were infected or not by RABV (CVS strain) and transfected or not by a plasmid encoding GFP-N. The cell supernatants were harvested, clarified, and centrifuged to pellet the viral particles. The pellet was analyzed by WB using anti-N and anti-P antibodies. No GFP-N was found in the pellet.*

*We agree with this remark. This is now presented in figure 4f. The transition occurs around 450 mM NaCl.*

6. To rigorously describe LLPS inhibition by RNAs, a phase diagram is required.

*In figure 6C, we did not study LLPS inhibition by RNAs. We wanted to investigate a potential induction of LLPS by moderate RNA concentration in absence of crowding agent (as this has been observed for some proteins in ref 29 and 30).*

*We acknowledge that the previous figure could be misleading. Accordingly, we have modified the panel 6C (previous 5C). We hope there is no more ambiguity.*

7. Lines 308-313 (fig 6h) Mixtures of P and NC could lead to observations of interest but only an image without quantification is presented.

*The image presented in figure 7h is just there to show that purified nucleocapsids do not enter into P droplets (spherical ones). We do not know what to quantify.*

8. Lines 314-318: Is RNA recruited to the droplets?

*We have added panel 7I to show that fluorescent RNA concentrates in N^0^P droplets.*

Can the concentration of RNA disassemble the condensates as before?

*Once again, RNA does not disassemble P droplets (see our answer to point 6).*

*However, it disrupts the spherical shape of N^0^P droplets as mentioned in the previous version of our manuscript.*

Are the results of the shape analysis different when RNAs are added before or at the same time as the crowding agent?

*We showed in the images presented in the previous figure 6I (now 7J) that we had a similar disruptive effect regardless of RNA addition times (10 minutes before PEG addition, at the time of PEG addition or 10 minutes after PEG addition).*

*We had quantified this effect in each condition. However, we had decided to present only the effect observed in the latter condition (addition of RNA 10 minutes after PEG addition) for better readability of the figure. Below is the full figure, the reviewer will see that, unsurprisingly, the disruptive effect of RNA is detected earlier when RNA is added at early times.*

*The legend of this figure is the same as for panel 7K. &&& reported a p-value <10^-6^ (calculated using Kolmogorov-Smirnov test) for the significance of the difference with droplets which were not incubated with RNA.*

**Reviewer #2:**

I would like to see OD280/260 ratios for the purified proteins to demonstrate that RNA contamination is not an issue inn the pure protein experiments. If this is in the methods and I missed it then apologies.

*We have now added an absorbance spectrum (figure S2B) which shows that the A260/A280 ratio is 0.59 which indicates that the protein is pure and uncontaminated with RNA.*

**Reviewer #3:**

This reviewer highlights some small points to address:

1- Figure 1 – The NB formed when co-expressing of N and P without infection are different in number and size relative to infection. The properties may be similar, but the characteristics of these NBs seem different relative to the concentration of material inside NBs and diffuse in the cytosol, which underlies the ability of material to phase separate. For example, in the controls GFP-N and P of Figures 1B, 1E and 1N, there is a relative high diffuse phase relative to concentrated in NBs for N, as compared to the low levels of diffuse N in infected cells at 14 h. The reasons for the differences are important to assess and absent in the manuscript. Would this be a matter of concentration? ), and assess Cdense/Cdiffuse by quantifying mean intensity, as in the manuscript "Composition-dependent thermodynamics of intracellular phase separation"
<https://www.nature.com/articles/s41586-020-2256-2.pdf>

The authors could test the levels of material by western blot in both conditions (co-expression and infection/transfection and test whether varying the concentration of either N, P and N and P results in different concentration of material inside and outside NBs.

2- By calculating Cdense and Cdiffuse as suggested above and by integrating the data with the western blots of S1C Fig and others, the authors would provide robust measurements taking into account cellular variation rather than stating in lines 180-181 “appeared to be higher than with wild-type N protein (as judged by the intensity of the cytoplasmic fluorescence intensity)”.

3- In figure 2B, the measurements of the concentration of N in the diffuse phase versus the dense phase would make the results robust and reveal biological properties of the condensates.

*We respond to these 3 points globally.*

*We fully agree that quantification is important. However, western blot gives an overall concentration and does not reflect intracellular variability in protein concentration. Therefore, as suggested by the reviewer, we have measured C_dense_ and C_diffuse_ for both P and N proteins in distinct cells. For this, we quantified the mean fluorescence in the diffuse phase and in the dense phase according to Riback et al. (ref 25) and assumed that it was proportional to the protein concentration in each phase.*

*These quantifications were performed both in the cellular minimal system (in which P was fused to the m-Cherry and N was fused to the GFP) and in cells infected by a recombinant RABV expressing a fluorescent version of P (P-mCherry) and transfected by a fluorescent version of N (a chimera between N and GFP).*

*All the quantifications are presented in figure 3 and in a novel paragraph (p11-12) in the results section. They confirm the difference in phenotypes of some mutants (such as those observed in Figs 1G and 2B). They are also discussed in a dedicated paragraph in the discussion (p20). Particularly, in the discussion we now wrote:*

*« This allowed us, for example, to demonstrate that the behaviors of GFP-N-R168A and GFP-N-R323A are distinct from those of GFP-N in the cellular minimal system and that they phase separate at higher P and N concentrations.*

*Nevertheless, the correlation between the concentrations of P and N in the dilute phases (simultaneously high or simultaneously low) is unexpected. This suggests that in many cases (when the dilute phase concentrations are high), we are far from equilibrium or that the state of the cell has a considerable influence on the phenomenon. In any case, the extraction of thermodynamic parameters from these data seems very difficult if not impossible. »*

4- P ability to undergo phase separation is different in cells or in P purified from E. coli -P purified from E. coli phase separates while in cells, P does not phase separate on its own. However, it is unknown whether P purified from cells can undergo phase separation. This distinction is important, as for example, it may suggest that P may contain post-translational modifications that abrogate phase separation. The purification of P from cells and associated studies on its ability to undergo phase separation are key to elucidate this point.

*We have expressed full-length P in Hi-5 insect cells using a recombinant baculovirus. This protein undergoes LLPS under conditions similar to P expressed in E.coli.*

**Reviewer #4:**

I did not find any problems

*We thank the reviewer for his/her overall agreement with our study.*

**Part III – Minor Issues: Editorial and Data Presentation Modifications**

**Reviewer #1:**

Minor points

1. Specify the FRAP protocol: (line 633) photobleaching is controlled fluorescence measurement in a neighboring cell. How is this region defined and is it a condensate? How is it corrected in vitro?

*We have completed the protocol. We now wrote: « To correct for acquisitional bleaching, the mean fluorescence measured in the bleached region was normalized by the mean value of a region in a neighboring cell (if possible a condensate) or in a nearby droplet (for the acellular system). »*

2. Fig1b, 1e and 1g: The condensates mentioned in the text are barely visible (especially in 1g). For Fig1b, a wild-type N + P control would be relevant.

*We added a wild-type N + P control (Figure 1B). On the original figures, the condensates are perfectly visible on a screen. We acknowledge that in the submitted pdf image quality is probably suboptimal. We hope that the submitted .tiff versions of the figures will correct this problem.*

3. Fig2d: How were the percentages of infected and transfected cells obtained? How many cells were analyzed for each experiment?

*The number of cells analyzed is now indicated in the figure legend.*

*The percentage of infected cells was obtained by dividing the number of infected cells (expressing P) by the number of nuclei in the field (X100).*

*The percentage of transfected cells was obtained by dividing the number of transfected cells (GFP positive) by the number of nuclei in the field (X100).*

*The percentage of infected and transfected cells was obtained by dividing the number of simultaneaously P and GFP positive cells by the number of nuclei in the field (X100).*

4. Hypotheses should be proposed or explored to explain the dominant negative effect of N-106GFP107 even though it is not recruited to NBs suggesting that it is not incorporated into the RNP.

*We do not want to speculate on this point. At this stage, many mechanisms could be proposed.*

5. Lines 245-247: The redistribution kinetics of P fluorescence are very different in cells (fig1c) and in droplets in vitro (fig3h) (even fresh). This point should be discussed.

*From our point of view, the major difference is that we are comparing an acellular system which reaches the equilibrium (and transitions toward a gel phase) versus a cellular system that remains dynamic. There is also a possible impact of post-translational modifications or the absence of cellular partners. In any case, this is consistent with other data in the literature.*

*These points were already mentioned all along the discussion in the paragraphs « Purified P and N^0^-P form liquid biocondensates in crowded environments » and « Final remarks ».*

6. For experiments mixing molecules with P or N0-P condensates, the molar ratio of the molecules brought together RNA versus P; lipids versus P, RNA versus N0-P, NC versus P should be given.

*In the figure legends, we had already indicated the concentration of each species except for BSA (3.8 µM) (Fig 6A), for the synthetic 40nt RNA used in figure 6C (6 µM), and for the lipids in the liposomes (~130µM) in figure 6D. The missing micromolar concentrations are now indicated in the respective panel legends. We let the reader calculate the molar ratios.*

7. An association between liposomes and P condensates is observed. Is it sensitive to the concentration of liposomes?

*It was difficult to perform this experiment over a wide range of liposome concentrations. We observed a similar association with 50 µg/ml of liposomes (i.e. ~65 µM of lipids)*

How does it evolve over time?

*The association is immediate and remains over a prolonged time (a few hours).*

8. Line 279, Fig5a: The RNA concentration (Fig5a) is not mentioned.

*The concentration (100nM) was indicated in the legend of the figure 6A (previously 5A). As suggested by the reviewer, this is now indicated in the text.*

The time of RNA addition is not specified (addition before droplet formation or on already formed droplets).

*Fluorescent RNA and BSA were added when LLPS was induced. Fluorescent RNA and BSA were added when LLPS was induced. This is now indicated in the figure legend.*

9. The units of RNA concentrations for Fig5b are in nM whereas they are in ng/µL in Fig5c.

*This has been changed (see above minor comment 6)*

**Reviewer #2:**

Perhaps the discussion could be sharpened a little to focus on new mechanistic findings and their relevance to viral replication

*After reading the body of comments, with some reviewers suggesting further discussion on very different points, we decided to keep our original discussion. Nevertheless, we add a short paragraph regarding quantifications because we believe that the conclusions we draw from this are important in the field.*

**Reviewer #3:**

Figure 2 – The levels of infected and transfected cells are very low. In the methods, the authors state that they infect and transfect 1h after infcetion. This may already activate an antiviral state in the infected cell. Have the authors attempted to infect and transfect at the same time to improve the efficiency of infected and transfected cells?

*In general, the level of transfection in BSR cells is low. These cells are known not to produce interferon. Thus, there is no antiviral state in these cells. This is also the reason why they are used very generally to produce viruses.*

*In fact, the cells were transfected 1h after adsorption (i.e. when the cells were transfered at 37°C). In the previous legend for figure 2A-2C, we had written 1h pi which was a bit confusing. We changed this to 1h post adsorption.*
